# Novel oligodendroglial alpha synuclein viral vector models of multiple system atrophy: studies in rodents and nonhuman primates

**DOI:** 10.1186/s40478-017-0451-7

**Published:** 2017-06-16

**Authors:** Ronald J. Mandel, David J. Marmion, Deniz Kirik, Yaping Chu, Clifford Heindel, Thomas McCown, Steven J. Gray, Jeffrey H. Kordower

**Affiliations:** 1 Department of Neurological Sciences, Rush University Medical Center, 1735 West Harrison Street, Chicago, IL 60611 USA; 2 Gainesville, University of Florida College of Medicine, po 100244, Gainesville, 32610 FL USA; 3 Department of Experimental Medical Science, Lund University, Lund, Sweden; 4 Gene Therapy Center, University of North Carolina, Chapel Hill, NC USA; 5 Department of Psychiatry, University of North Carolina, Chapel Hill, NC USA; 6 Department of Ophthalmology, University of North Carolina, Chapel Hill, NC USA; 7 Van Andel Institute, Grand Rapids, MI USA

**Keywords:** Multiple system atrophy, Alpha synuclein, Nonhuman primate, Adeno-associated virus

## Abstract

Multiple system atrophy (MSA) is a horrible and unrelenting neurodegenerative disorder with an uncertain etiology and pathophysiology. MSA is a unique proteinopathy in which alpha-synuclein (α-syn) accumulates preferentially in oligodendroglia rather than neurons. Glial cytoplasmic inclusions (GCIs) of α-syn are thought to elicit changes in oligodendrocyte function, such as reduced neurotrophic support and demyelination, leading to neurodegeneration. To date, only a murine model using one of three promoters exist to study this disease. We sought to develop novel rat and nonhuman primate (NHP) models of MSA by overexpressing α-syn in oligodendroglia using a novel oligotrophic adeno-associated virus (AAV) vector, Olig001. To establish tropism, rats received intrastriatal injections of Olig001 expressing GFP. Histological analysis showed widespread expression of GFP throughout the striatum and corpus callosum with >95% of GFP+ cells co-localizing with oligodendroglia and little to no expression in neurons or astrocytes. We next tested the efficacy of this vector in rhesus macaques with intrastriatal injections of Olig001 expressing GFP. As in rats, we observed a large number of GFP+ cells in gray matter and white matter tracts of the striatum and the corpus callosum, with 90–94% of GFP+ cells co-localizing with an oligodendroglial marker. To evaluate the potential of our vector to elicit MSA-like pathology in NHPs, we injected rhesus macaques intrastriatally with Olig001 expressing the α-syn transgene. Histological analysis 3-months after injection demonstrated widespread α-syn expression throughout the striatum as determined by LB509 and phosphorylated serine-129 α-syn immunoreactivity, all of which displayed as tropism similar to that seen with GFP. As in MSA, Olig001-α-syn GCIs in our model were resistant to proteinase K digestion and caused microglial activation. Critically, demyelination was observed in the white matter tracts of the corpus callosum and striatum of Olig001-α-syn but not Olig001-GFP injected animals, similar to the human disease. These data support the concept that this vector can provide novel rodent and nonhuman primate models of MSA.

**Electronic supplementary material:**

The online version of this article (doi:10.1186/s40478-017-0451-7) contains supplementary material, which is available to authorized users.

## Introduction

Multiple system atrophy (MSA) is a fatal neurodegenerative disorder that presents clinically with varying combinations of autonomic, parkinsonian, cerebellar, and pyramidal dysfunction [15]. It is considered to be an orphan disease, with an estimated mean incidence of 0.6–0.7 cases per 100,000 person-years [5]. Symptoms tend to emerge in the fifth to sixth decade of life [63], and unlike Parkinson’s disease (PD) progresses extremely rapidly, with a mean survival of 6–9 years following symptom onset [35, 41, 50, 62]. The distribution of pathology allows for stratification into two MSA subtypes, a cerebellar variant (MSA-C), characterized by olivopontocerebellar atrophy, and a parkinsonian variant (MSA-P), characterized by nigrostriatal and striatonigral degeneration [39, 65]. MSA, like PD, involves a prodromal premotor phase, with symptoms including urinary incontinence or retention, sexual dysfunction, orthostatic hypotension, and rapid-eye-movement sleep behavior disorder [27]. Following the prodromal phase, motor symptoms in MSA-P develop, including slowness of movement, rigidity, postural instability, and shuffling gait [18, 32, 38, 60]. Lack of “pill-rolling” rest tremor, symmetrical presentation of symptoms, and limited levodopa responsiveness can often distinguish MSA-P from PD [18, 32, 38, 60].

MSA is a synucleinopathy, however, unlike PD and dementia with Lewy bodies, in which α-syn accumulates and aggregates in neurons, MSA is characterized neuropathologically by α-syn-positive glial cytoplasmic inclusions (GCIs) in oligodendroglia [1, 42, 54, 58, 59]. The precise pathogenesis of MSA remains largely unknown, however, evidence from human postmortem cases and experimental models suggest that, in addition to GCIs, myelin degeneration, axonal damage, loss of neurotrophic support, neuronal loss, astrogliosis and microglial activation are pathological events seen in multiple regions of the central nervous system (CNS) [28, 56]. The primary cause of degeneration is still under debate- whether cell loss in MSA is secondary to disruptions in the oligo-myelin-axon complex [29, 64], or if MSA is a primary neuron disease, with the secondary formation of GCIs following pathological accumulation of α-syn that is neuronal in origin [55]. Experimentally, α-syn has been shown to transfer from neurons to oligodendrocytes in vitro [47], and inoculation of MSA brain homogenates into transgenic mice supports the hypothesis of prion-like spread in MSA [45, 61]. However, formation of GCI-like aggregation was not reported in these transfer experiments [47]. Supporting a primary oligodendrogliopathy, GCIs, and not neuronal inclusions, are a pathology first observed in regions where significant neurodegeneration occurs, with GCI density correlating with the degree of neuron loss [40, 43, 68]. Moreover, GCIs are the hallmark of MSA and are not seen as frequently in PD, even though both diseases share similar lesion patterns in many overlapping circuits [23].

Transgenic (tg) mouse models overexpressing human α-syn under different oligodendroglia-specific promoters, such as proteolipid protein (PLP) [31], myelin basic protein (MBP) [51], and 2′,3′-cyclic nucleotide 3′-phosphodiesterase (CNP) [67], have been developed to study MSA. The resulting tg mouse lines developed widespread GCIs, however varying degrees of demyelination, neurodegeneration, and behavioral changes have been reported [4, 16, 31, 33, 50–53, 67]. To date, none of the available tg mouse lines have been able to recapitulate the specific striatonigral degeneration or olivopontocerebellar atrophy as seen in the human disorder [3].

Adeno-associated virus (AAV) has been successfully used to overexpress α-syn in dopaminergic neurons to create useful animal models to study PD in rodents and nonhuman primates [19, 57]. AAV is a small, encapsulated parvovirus with a simple genome encoding 2 genes for packaging and replication. In 1984, Hermonat and Muzyczka showed that the entire genome could be removed and DNA could be packaged in replication deficient AAV, allowing for the transfer genes to be ectopically expressed in transduced cells [24]. A number of AAV capsid mutants have been created, leading to improvements in target cell specificity, efficiency of transduction, and reduced immunogenicity [22]. In the CNS, most wild-type AAV capsid serotypes transduce neurons at a much higher rate than any other neural cell type, making it difficult to manipulate glial cells. Moreover, the AAV capsid cell tropisms obtained in rodents are not always predictive of the transduction specificity in primates. For example, in our own work, AAV1, 5 and 8 almost exclusively transduce neurons in rat striatum whereas, in the macaque putamen AAV1, 5, and 8 transduced glial cells at higher rates [11].

In an effort to develop a viral-vector mediated model of MSA, we utilized a novel AAV capsid termed Olig001 and developed by co-authors in our group (SG and TM). The vector was engineered by capsid shuffling and directed evolution to transduce oligodendrocytes in the striatum and corpus callosum of rodents [44]. In the present study, we showed that 4-weeks following intrastriatal injection of Olig001 expressing GFP transgene leads to overwhelming oligodendroglia-specific tropism in both rats and monkeys, with little to no expression in neurons or astrocytes. We then used this vector to overexpress human α-syn in the striatum and corpus callosum of rhesus monkeys. After 3-months, we observed widespread expression of α-syn in the white matter of the striatum, again overwhelmingly within oligodendroglia. These α-syn GCIs were phosphorylated at serine-129 (pSer-129), resistant to proteinase K (PK) digestion, resulted in demyelination in the striatum and corpus callosum and activated microgla in the substantia nigra. Together, these data indicate the development of the first pathologically-based nonhuman primate model of MSA-P.

## Materials and methods

### AAV vector

The AAV-Olig001 capsid was developed in the laboratories of Steven Gray and Thomas McCown at the University of North Carolina at Chapel Hill, through a directed evolution screening process as described [20, 44]. Preliminary studies demonstrated a strong preferential tropism of this capsid for oligodendrocytes following intracranial administration in rodents (data not shown). The AAV-Olig001 vector used in these studies packaged a self-complementary (sc) genome with transgene expression mediated by the CBh promoter and bovine growth hormone polyA [21]. AAV vectors were produced using methods developed by the University of North Carolina Vector Core facility, as described [9]. In brief, the production plasmids (pXX6–80, pTRS-ks-CBh-EGFP, and AAV pXR-Olig001) were triple-transfected into suspension HEK293 cells. AAV vectors were purified from the cells by iodixanol gradient centrifugation, followed by ion-exchange chromatography. The purified AAV was dialyzed in PBS supplemented with 5% D-Sorbitol and an additional 212 mM NaCl (350 mM NaCl total). The titer was determined by quantitative PCR and confirmed by polyacrylamide gel electrophoresis (PAGE) and silver stain. Quality control measures were in place that the qPCR titer and PAGE/silver stain titer match within 2-fold, that no contaminating proteins are visible by PAGE, and that the viral capsid proteins migrate at the expected size with a 10:1:1 VP3:VP2:VP1 ratio.

### Rodents

Eight-week-old female Sprague-Dawley rats (Harlan, Indianapolis, IN) were used for the rodent rAAV-Olig001-GFP experiment. Upon arrival, animals were quarantined for one week prior to any testing. Animals were cared for in accordance with the principles of the Guide to the Care and Use of Experimental Animals, and all procedures were approved by University of Florida at Gainesville Institutional Animal Care and Use Committee. Rats were housed two per cage with a 12:12 h light:dark cycle (07:00–19:00 h). Food and water were available ad libitum throughout the study.

### Rodent stereotaxic surgery

All surgical procedures were performed as previously described [36]. All animals received unilateral striatal injections 15 min after receiving intraperitoneal injections of mannitol (3 ml sterile 25% mannitol in 0.9% saline / 100 g body weight). Each rat received 1 injection of 2 μl of Olig001-GFP (1 × 10^13^ vg/ml) at a rate of 0.5 μl/min [6]. The coordinates were AP +/− 0.0 mm and ML +/− 2.7 mm from bregma, DV −4.0 mm from dura. One minute following the completion of each injection the micropipette was retracted 1 mm and left in place for an additional 4 min before being slowly removed from the brain.

### Rodent necropsy and tissue processing

Four-weeks following the intracerebral rAAV-Olig001 injection all animals were deeply anesthetized with pentobarbital and perfused through the ascending aorta with sterile Tyrode’s solution, followed by 350 ml of ice-cold 4% paraformaldehyde in 0.01 M PBS buffer. Brains were rapidly removed and post-fixed for 12–18 h in the same paraformaldehyde solution, and then transferred to a 30% sucrose in 0.01 M PBS solution. After sinking in the sucrose solution, the brains were cut into 40 μm thick sections using a freezing stage sliding microtome and then processed for immunocytochemistry as described below.

### Primates

Sixteen nonhuman primates (eight adult male and eight adult female rhesus monkeys (*Macaca mulatta*) served as subjects and/or were analyzed for this study. Monkeys were placed in 4 groups of three monkeys each; Group 1: Olig001-GFP sacrificed at 1 month, Group 2: Olig001-α-syn sacrificed at 3 months; Group 3: intrastriatal AAV injections of neurturin sacrificed at 3 months; Group 4: intrastriatal stem cell grafted animals sacrificed at 3 months. Group 5: untreated control.

Since the Olig001-GFP (1 month) and Olig001-α-syn (3 months) were sacrificed at different time points, and to minimize the number of monkeys required for experimentation, Groups 3 and 4 were historical controls in one of our labs (JHK) and served to control for the effects of 1) anesthesia, 2) surgery, 3) needle penetration to the striatum, and delivery of 4) AAV and 5) other bioactive bioactive materials and all were sacrificed at the same post-operative time as Olig001-α-syn treated animals.

Animals were pair-housed on a 12-h light/12-h dark cycle. All procedures were approved by the University of Illinois Chicago Institutional Animal Care and Use Committee and the Rush University Institutional Animal Care and Use Committee and accredited by the Association for Assessment and Accreditation of Laboratory Animal Care. Animal care was supervised by veterinarians skilled in the care and maintenance of nonhuman primates.

### Primate stereotaxic surgery

Animals were tranqilized and then intubated on the day of surgery with ketamine (10 mg/kg, IM) and propofol (2–5 mg/kg, IV) and placed in sterotaxic frames in the surgical suite. All surgical procedures were conducted under isoflurane anesthesia (1–3% maintenance, inhalation) and sterile field conditions. Sufentanil (0.005–0.3 μg/kg/min, IV infusion) or hydromorphone (0.05–0.2 mg/kg, IV) was administered pre-operatively, as was Cefazolin (25 mg/kg, IV). Surgical targets were identified using pre-operative MRI and intraoperative surgical navigation using a Stealthstation Neuronavigation system (generously donated by Medtronics Inc.). A 50-μl Hamilton syringe fitted with a 22 G needle was loaded with either Olig001-GFP (1 × 10^13^ vg/ml) or Olig001-α-syn (3.75 × 10^12^ vg/ml). Animals received injections unilaterally into the putamen (rostral putamen 10 μl, caudal putamen 5 μl) and caudate nucleus (rostral caudate 10 μl, caudal caudate 5 μl) and infused at a rate of 1 μl/min to minimize injectate reflux, inflammation or damage to the parenchyma. After the injection, the needle was left in place for 2 min, then slowly retracted. Bupivicane (1 mg/kg, SQ) was administered to the incision site prior to closure. Animals received analgesics (Buprenex SR, 0.2 mg/kg SQ, once post surgery; Meloxicam, 0.2 mg/kg SQ, once post surgery, then 0.1 mg/kg SQ, SID for 2 days post surgery) and antibiotics (Cephazolin, 25 mg/kg IM, BID for 3 days post surgery).

### Primate necropsy and tissue processing

One or three-months post-surgery, animals were deeply sedated and euthanized by transcardial saline perfusion. The brain was removed from the calvarium and was post-fixed in 4% paraformaldehyde solution for 48 h then transferred to a sucrose gradient. Coronal slices (40 μm) were sectioned on a freezing-stage sliding knife microtome, and stored in cryoprotectant solution at 20 °C until processed.

### Immunohistochemistry

Free floating sections of brain tissue were rinsed of cryoprotectant and quenched with endogenous peroxidases using a 20-min incubation in a 0.1 M sodium periodate solution. Non-specific background staining was blocked for 1 h in 3% normal (goat or horse) serum and 2% bovine serum albumin. Sections were incubated with primary antibody (mouse anti-Living Colors JL-8 (GFP), 1:2000 dilution [632,380; Clontech]; rabbit anti-Alpha-synuclein (phospho S129), 1:500–1:1000 dilution [ab51253; Abcam]; mouse anti-Alpha-synuclein (LB 509), 1:500–1:1000 dilution [180,215; Thermo Fischer Scientific]; mouse anti-HLA-DR (LN3), 1:200 dilution [MA5–11966, Thermo Fischer Scientific]), 1% bovine serum albumin, 1% serum, and 0.4% Triton-X at 4 °C for 18 h. The sections were then washed, incubated with appropriate secondary antibodies (biotinylated goat anti-rabbit, 1:200 dilution [BA-1000, Vector Laboratories]; biotinylated horse anti-mouse, 1:200 dilution [BA-2000, Vector Laboratories]) for 1 h, washed again, and incubated with avidin-biotin complex (Vector Laboratories, PK-6100) for 2 h. Tissues were then incubated in imidazole-acetate buffer, pH 7.3, for 30 min before they were visualized with 3–3′-diaminobenzidine tetrahydrochloride in 0.01% hydrogen peroxide with 2% nickel enhancement. The sections were allowed to dry overnight, dehydrated through increasing alcohol concentrations and xylenes, and coverslipped with cytoseal (23,244,257; Fisher Scientific International).

### Immunofluorescence double labeling

Free floating sections of brain tissue were rinsed of cryoprotectant and non-specific background staining was blocked for 1 h in 3% normal (goat) serum and 2% bovine serum albumin. Sections were incubated with primary antibody (mouse anti-Living Colors JL-8 (GFP), 1:2000 dilution [632,380; Clontech]; mouse anti-Alpha-synuclein (LB 509), 1:500–1:1000 dilution [180,215; Thermo Fischer Scientific]; rabbit anti-Olig2 [EPR2673], 1:200 dilution [ab109186; Abcam]; rabbit anti-GFAP, 1:2000 dilution [Z033401–2; Dako]; mouse anti-NeuN (A60), 1:500 dilution [MAB 3777; EMB Millipore]), 1% bovine serum albumin, 1% serum, and 0.4% Triton-X at 4 °C for 18 h. The sections were then washed, incubated with appropriate secondary antibodies (Alexa Fluor® 488 AffiniPure Goat Anti-Mouse IgG, 1:200 [115–545-003; Jackson Immuno Research Laboratories]; Alexa Fluor® 647 AffiniPure Fab Fragment Goat Anti-Mouse IgG, 1:200 [115–607-003; Jackson Immuno Research Laboratories]; Alexa Fluor® 488 AffiniPure Goat Anti-Rabbit IgG, 1:200 dilution [111–545-003; Jackson Immuno Research Laboratories]; Alexa Fluor® 647 AffiniPure Goat Anti-Rabbit IgG, 1:200 dilution [111–605-003; Jackson Immuno Research Laboratories]) for 1 h, washed again, and mounted on glass slides. The sections were allowed to dry overnight, dehydrated through increasing alcohol concentrations and xylenes, and coverslipped with DPX mounting medium (Sigma, 44,581).

### Proteinase K digestion

PK digestion was used to determine whether the α-syn seen in oligodendrocytes was soluble (non-aggregated) or insoluble (aggregated). Striatal sections containing the injection site were mounted onto gelatin-coated slides and dried for at least 8 h at 55 °C. After wetting with TBS-T (10 mM Tris–HCl, pH 7.8; 100 mM NaCl; 0.05% Tween-20), the sections were digested with 10 μg/ml PK (Invitrogen) in TBS-T (10 mM Tris–HCl, pH 7.8; 100 mM NaCl; 0.1% Tween-20) for 30 min at 55 °C. The sections were fixed with 4% paraformaldehyde for 10 min. After several washes, the sections were processed for α-syn immunostaining as described above.

### Luxol Fast Blue

For analysis of myelin integrity, level matched sections of both Olig001-GFP and Olig001-α-syn injected monkeys were mounted on glass slides and allowed to dry overnight. Sections were placed in a 1:1 alcohol/chloroform solution to defat the tissue, rinsed in 70% alcohol, and placed in a 0.1% Luxol Fast Blue solution overnight at 56 °C. Sections were destained using 0.05% lithium carbonate solution and 70% alcohol until gray matter was clear and white matter was clearly defined.

### Stereology

In order to estimate the number cells transduced by Olig001, GFP+ or pSer-129+ cells in rats and monkeys in an unbiased fashion, stereological counting methods [66] using the optical fractionator probe in Stereo Investigator (Microbrightfield Bioscience, Version10.40) were applied. A random and systematic sampling of sections were employed in both rats and monkeys with every 12th section being analyzed. Eight equispaced sections per rat and 20 equispaced sections per monkey were evaluated. The transduction area in the striatum was outlined using a 1.25× objective. Cells were counted from a random starting point at regular predetermined intervals (*x* = 300 μm, *y* = 300 μm) with a counting frame (70 μm × 70 μm) using a 60× oil immersion objective. The coefficients of error (CE) were calculated according to the procedure of Gunderson and colleagues as estimates of precision [66]. The values of CE were 0.047 ± 0.006667 (Gunndersen m = 0) for pSer-129+ cells and 0.063 ± 0.012 (Gunndersen m = 0) for GFP+ cells in NHPs, and 0.105 ± 0.0087 GFP+ cells for rats. The volume of the striatum occupied by the transduced cells was also measured and recorded using the Stereo Investigator optical fractionator probe in the same manner by which the cells were counted. To assess the specificity of transduction and to determine any potential off-target transduction, double-label immunofluorescence was employed, as described above, using GFP combined with Olig2, NeuN, or GFAP. The percentage of double-labeling was determined by counting GFP transduced cells in the striatum of rats and nonhuman primates using the Stereo Investigator optical fractionator probe. Serial sections of the entire rostral-caudal striatum containing the transduction area were used, counting every 12th section (8 sections counted per rat, 20 sections counted per monkey).

### Images

Confocal images were exported from the Olympus laser-scanning microscope with Fluoview software and stored as tiff files. Conventional light microscopic images were acquired using an Olympus microscope (BX61) attached to a Nikon digital camera DXM1200 and stored as tif files. All compared images were taken using the same intensity and exposure time. All figures were prepared using Photoshop 8.0 graphics software. Only minor adjustments of brightness were made.

## Results

### Intrastriatal injection of Olig001-GFP produces widespread and specific transduction of oligodendroglia in rats

**Fig. 1:**
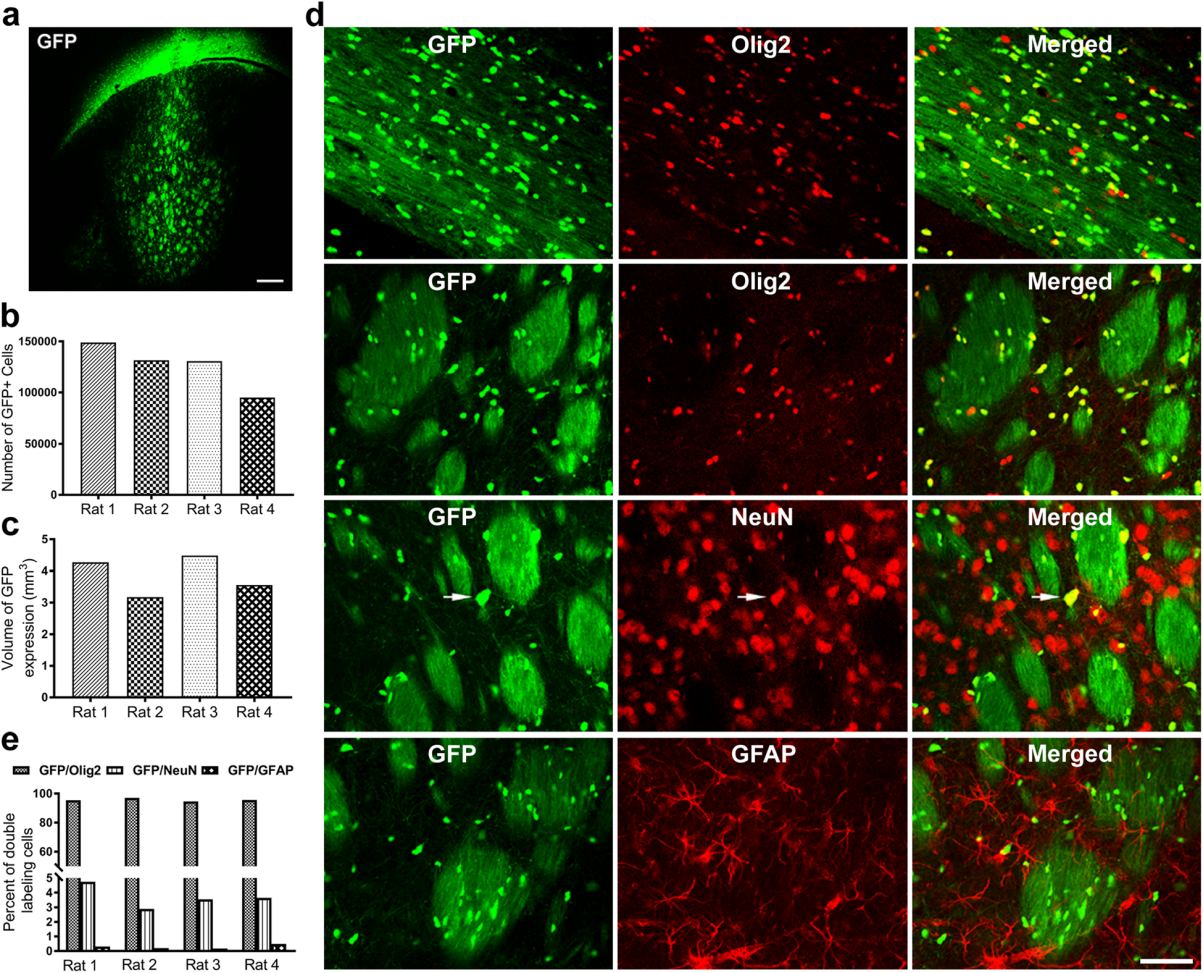
Olig001-GFP expression in rats. **a** Representative image of GFP expression in the striatum and corpus callosum of rats 4-weeks following injection of Olig001-GFP. Robust GFP expression is seen in the white matter bundles in the striatum and corpus callosum (*n* = 4). *Scale bar* 100 μm. **b** Stereological quantification estimated 126,574 ± 11,303.77 GFP+ cells, **c** which occupied a volume of 3.873 ± 0.304 mm^3^ in the striatum. **d** GFP expression is seen to colocalize with oligodendroglia marker Olig2 in the corpus callosum (top panel) and striatum (second panel). Very little GFP expression is seen to colocalize with neuronal marker NeuN (third panel) or astrocyte marker GFAP (bottom panel). *Scale* bar 50 μm. **e** 94–97% of GFP+ cells in the corpus callosum and striatum co-localized with oligodendrocyte marker Olig2, only 2.9–4.7% of GFP+ cells co-localizing with neuronal marker NeuN, 0.18–0.49% of GFP+ cells co-localizing with astrocyte marker GFAP phenotype [effect of phenotype F(2,9) = 11,711.6, *P* < 0.0001, oligodendrocytes vs. astrocytes *p* < 0.0001 Fisher’s PLSD post hoc, oligodendrocytes vs. neurons, *p* < 0.0001, Fisher’s PLSD, astrocytes vs. neurons *p* = 0.0001 Fisher’s PLSD]

In an effort to develop a novel viral vector mediated model of MSA, we utilized a AAV capsid, Olig001, that was previously shown to exhibit a selective tropism for striatal oligodendrocytes [44]. To assess the effectiveness of Olig001 to transduce oligodendrocytes with GFP, rats received a single 2 μl stereotaxic injection of Olig001-GFP (1 × 10^13^ vg/ml) in the striatum. As expected, histological analysis 4-weeks following injection of Olig001-GFP revealed widespread transduction of cells, as shown by intense GFP+ fluorescence in the dense white matter of the corpus callosum and white matter bundles coursing the striatum in all injected animals (Fig. 1a). Quantification of GFP+ cells in serial sections throughout the rostrocaudal striatum using unbiased stereological cell counting methods estimated that 126,574 ± 11,303 cells in the striatum and corpus callosum were GFP+ (Fig. 1b). We also quantified the spread of the injected vector, and found that GFP+ cells occupied a volume of 3.873 ± 0.304 mm^3^ in the striatum (Fig. 1c).

Morphologically, the expression pattern of GFP+ cells appeared to be specific to oligodendrocytes, consistent with the oligodendroglial-specific tropism of Olig001, as the GFP signal was strong in the oligo-rich white matter of the corpus callosum, with distinct labeling of myelin in the patch component of the striatal patch-matrix (Fig. 1a). In order to conclusively determine the specificity of transduction, and to evaluate any potential non-oligodendroglial tropism, we performed quantitative immunofluorescence double labeling experiments to determine the specific phenotype of transduced cells. In independent analyses, native GFP expression was coupled with Olig2, as a marker for oligodendrocytes, NeuN, as a marker for neurons, or GFAP, as a marker for astrocytes. Stereological quantification indicated that 94–97% of GFP+ cells in the corpus callosum and striatum co-localized with oligodendrocyte marker Olig2, indicating that Olig001 transduction was highly specific for oligodendroglia (Fig. 1d top two panels). Conversely, there was very little off target transduction of neurons, with only 2.9–4.7% of GFP+ cells co-localizing with neuronal marker NeuN (Fig. 1d third panel), and virtually no transduction of astrocytes, with only 0.18–0.49% of GFP+ cells co-localizing with astrocyte marker GFAP (Fig. 1d bottom panel). The quantitative data are shown in Fig. 1e for each cellular phenotype [effect of phenotype F(2,9) = 11,711.6, *P* < 0.0001, oligodendrocytes vs. astrocytes *p* < 0.0001 Fisher’s PLSD post hoc, oligodendrocytes vs. neurons, *p* < 0.0001, Fisher’s PLSD, astrocytes vs. neurons *p* = 0.0001 Fisher’s PLSD]. Confocal microscopic analysis demonstrated that GFP+ cells co-existed inside of oligodendrocytes as shown by z-stack images (Additional file 1: figure S1). Taken together, these data demonstrate the effectiveness of Olig001 to transduce oligodendroglia in the rat striatum and highlight its oligo-specific tropism.

### Intrastriatal injection of Olig001-GFP produces widespread and specific transduction of oligodendroglia in nonhuman primates

**Fig. 2:**
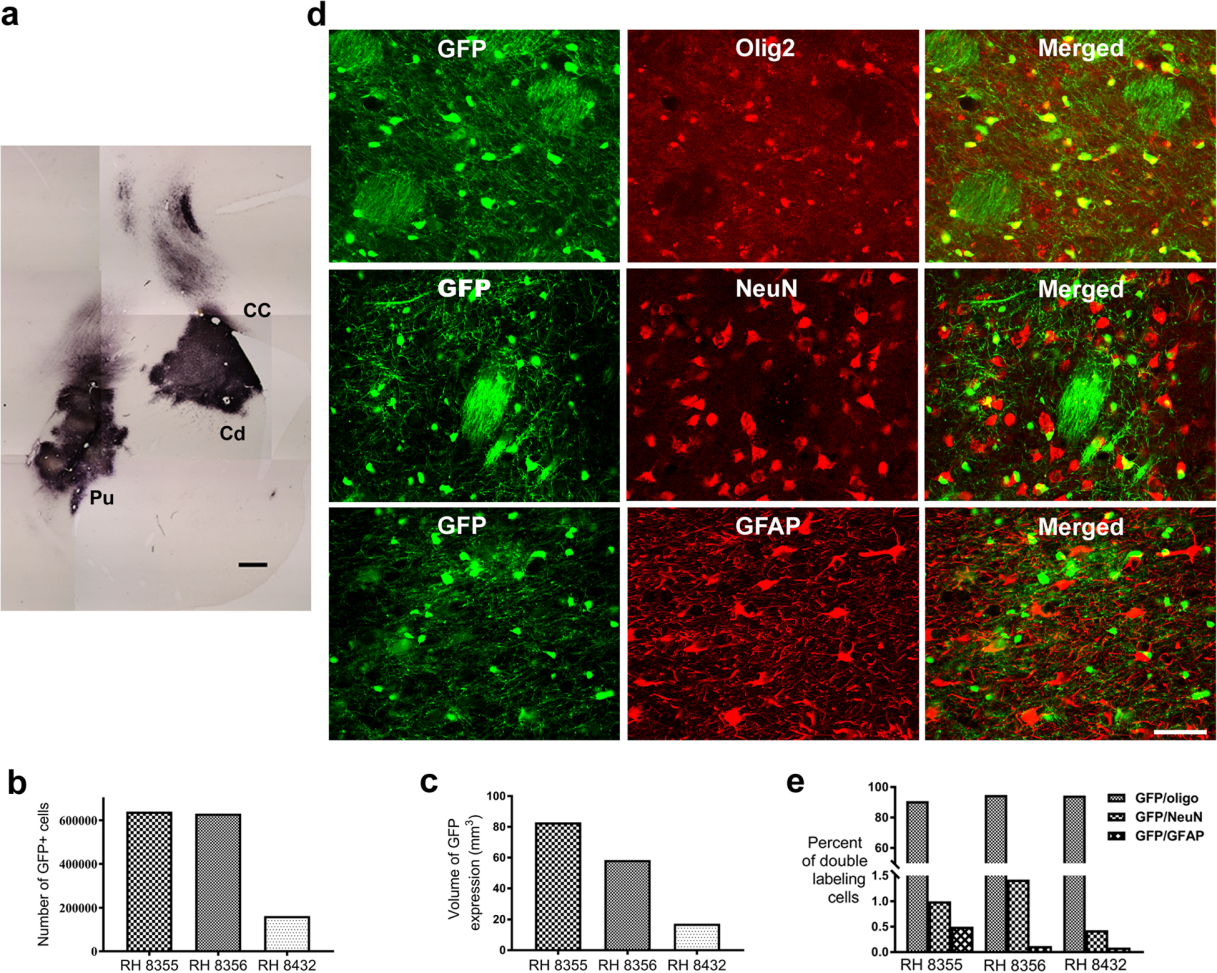
Olig001-GFP in NHPs **a** Representative image of GFP expression in the striatum and corpus callosum of rhesus macaques 4-weeks following injection of Olig001-GFP. Robust GFP expression is seen in the white matter bundles in the striatum and corpus callosum (*n* = 3) (CC corpus callosum, Cd caudate, Pu putamen, *Scale bar* 1 mm). **b** Stereological cell counts revealed an estimated 477,448.33 ± 157,773.29 GFP+ cells, **c** which had a transduction volume of 52.89mm^3^ ± 19.17 mm^3^. **d**,**e** Double labeling experiments show that 90–94% of GFP+ cells (*green*) co-localized with Olig2 (*red*) (*top panel*), and only 0.23–1.42% of GFP+ cells co-localized with NeuN (*red*, *middle panel*), and only 0.06–0.12% co-localized with GFAP (*red*, *bottom panel*). *Scale bar* 50 μm

Given the ability of Olig001 to preferentially transduce oligodendroglia with GFP in the rodent brain, we next evaluated the efficacy of Olig001 to transduce oligodendrocytes in the NHP brain. The same vector construct was injected into 3 adult rhesus macaques (*Macaca mulatta*). In concurrence with the results from our rat transduction experiment, 4-weeks following intracerebral injection of Olig001-GFP, histological analysis displayed a large number of GFP+ cells in the primate caudate, putamen, and corpus callosum (Fig. 2a). Stereological cell counts revealed an estimated 477,448.33 ± 157,773.29 GFP+ cells (Fig. 2b), which had a transduction volume of 52.89 mm^3^ ± 19.17 mm^3^ (Fig. 2c).

We next performed immunofluorescence double labeling experiments to determine the transduction profile of Olig001 in the NHP brain, and to evaluate if there were any differences in tropism between species. As with the rats, stereological quantification indicated that Olig001 demonstrated a marked oligodendroglial-specific tropism in the NHP brain, with 90–94% of GFP+ cells co-localized with oligodendrocyte marker Olig2 (Fig. 2d, top panel). Remarkably, Olig001 produced virtually no off target transduction of neurons or astrocytes in the primate brain, with only 0.23–1.42% of GFP+ cells co-localized with NeuN, and only 0.06–0.12% co-localized with GFAP (Fig. 2d, middle and bottom panel, respectively). Although one monkey had far fewer transduced cells and a much smaller volume of transduction (RH 8355 Fig. 2b, c), Olig001 displayed oligodendrocyte specific tropism in all animals injected. These data indicate a strong preference of Olig001 to transduce oligodendrocytes in NHP brain [effect of phenotype F(2,6) = 4788.2 *p* < 0.0001, oligodendrocytes vs. astrocytes *p* < 0.0001 Fischer’s PLSD post hoc, oligodendrocytes vs. neurons *p* < 0.0001 Fischer’s PLSD post hoc, neurons vs. astrocytes, *p* > 0.68, Fig 2e]. Confocal microscopic analysis demonstrated that GFP+ cells co-existed inside of NHP oligodendrocytes as shown by z-stack images (Additional file 2: figure S2).

Moreover, there was no difference between the phenotypic transduction of oligodendrocytes in rodents and primates [F(1,5) = 3.4, *p* > 0.12]. The percentage of co-expression of GFP and GFAP to characterize astrocytic transgene expression was also not different between rats and NHPs [F(1,5) = 5.3, *p* > 0.06]. In contrast to glial co-expression, there was slightly more co-expression of GFP and NeuN in rodent striatum and corpus callosum than in similar brain regions in NHPs [3.7% rodents vs. 0.7% primates (F1,5) = 31.7, *p* = 0.002], indicating that Olig001 produced less off target transduction in nonhuman primates (Additional file 3: figure S3).

### Intrastriatal injection of Olig001-α-syn produces widespread and specific transduction of oligodendroglia in nonhuman primates

Knowing the ability of Olig001-GFP to successfully transduce a large number of cells and demonstrate highly specific oligodendroglia tropism, we next wanted to transduce NHP oligodendroglia with human α-syn, to begin to establish the first NHP model of MSA. To do so, rhesus macaques were stereotactically injected with Olig001-α-syn (3.75 × 10^12^ vg/ml) unilaterally into the caudate and putamen. In an effort to follow the “3 Rs” of IACUC, AAV injected, stem cell grafted monkeys and untreated monkeys were utilized from previous studies to serve as control groups for the 3-month time point. No oligodendroglial tropism, expression of α-syn, or demyelination were seen in any of these control animals and thus they were not included in any statistical analyses (data not shown).

**Fig. 3:**
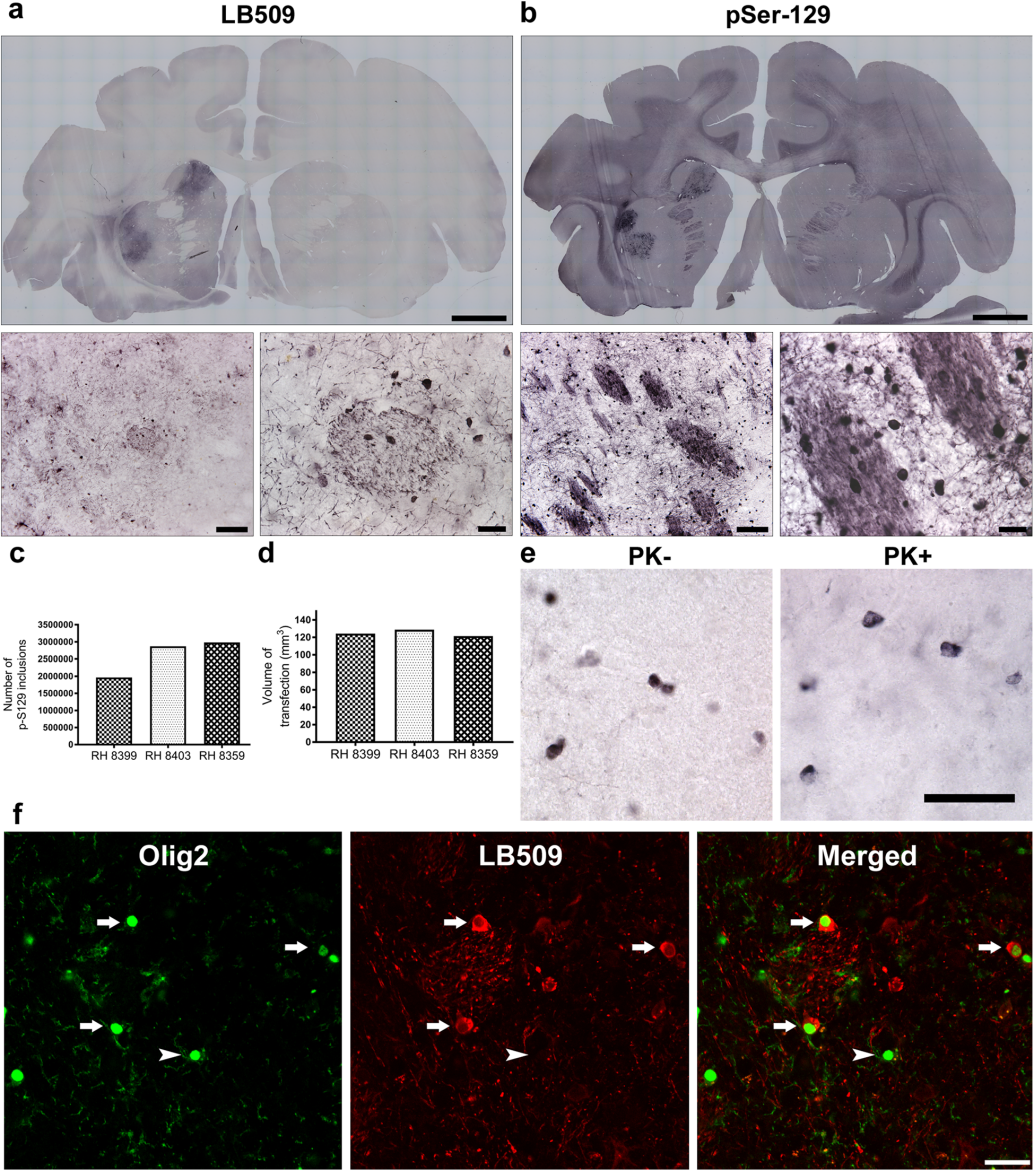
α-Syn Expression in NHPs Following injection of Olig001-α-syn. Representative **a** LB509 and **b** phosphorylated serine-129 stained image striatal sections of nonhuman primate brains 3-months following administration of Olig001-α-syn (*scale bar* 5 mm) . α-syn is seen throughout the striatum in white matter tracts (high magnification, *scale bar* 100 μm, 25 μm) **c** Quantification using unbiased stereological counts of pSer-129 inclusions estimates 2,606,199.5 ± 322,082.98 pSer-129+ aggregates, **d** which covered 124.8 ± 2.18 mm^3^ of the striatum. **e** pS129 staining with and without proteinase K digestion indicates the formation of insoluble aggregates throughout the striatum (*scale bar* 30 μm). **f** Confocal microscopy confirms the cytoplasmic localization of α-syn in oligodendrocytes, shown by LB509 immunoreactivity (*red channel*) wrapped around nuclear Olig2 signal (*green channel*) (*scale bar* 300 μm)

Histologically, 3-months following injection of Olig001-α-syn, robust soluble and aggregated α-syn was seen throughout the caudate, putamen, and corpus callosum of all 3 primates, as shown by LB509 (Fig. 3a) and pSer-129 α-syn immunoreactivity (Fig. 3b). Expression patterns of α-syn were similar to that of GFP, where α-syn was seen in white matter patches throughout the striatum, indicative of oligodendroglial-specific transduction (Fig. 3a and b high magnification). Quantification using unbiased stereological counts of pSer-129 inclusions estimates 2,606,200 ± 322,083 pSer-129+ aggregates (Fig. 3c), which covered 124.8 ± 2.18 mm^3^ of the striatum (Fig. 3d). No α-syn expression was seen in untreated or stem cell grafted control groups.

Morphological analysis suggested that the α-syn expression was restricted to oligodendrocytes, as inclusions were prominent in the corpus collosum and white matter bundles coursing within the striatum (Fig. 3a and b high magnification). To evaluate the specific localization of α-syn inclusions, immunofluorescence double labeling was performed using LB509 and Olig2. As a technical note, we used LB509 and not pSer-129 as a marker for α-syn because the olig2 and pSer-129 antibodies were generated in the same species. Confocal microscopy confirmed the cytoplasmic localization of α-syn inside oligodendroglia (Fig. 3f), indicating that our model develops GCIs, the pathological hallmark of MSA. Moreover, the morphology of the GCIs (Fig. 3f, red channel) mimics the typical conical half-moon shape inclusions seen in the human disease [43]. α-syn GCIs in MSA are known to be insoluble and phosphorylated at serine residue 129 [17], so to further characterize the α-syn inclusions seen in our model, PK digestion was used to determine whether the Olig001-induced overexpression of α-syn lead to the formation of soluble (non-aggregated) or insoluble (aggregated) inclusions. Following PK treatment, abundant pSer-129 immunoreactive α-syn inclusions remained apparent throughout the striatum of Olig001-α-syn injected monkeys (Fig. 3e), indicating the formation of insoluble GCIs, as seen in MSA patients. Taken together, these indicate that Olig001 successfully transduces NHP oligodendroglia with α-syn, leading to the accumulation of widespread insoluble, pSer-129 immunoreactive GCIs, recapitulating pathological characteristics MSA.

### Olig001-α-syn expression leads to demyelination in NHPs

**Fig. 4:**
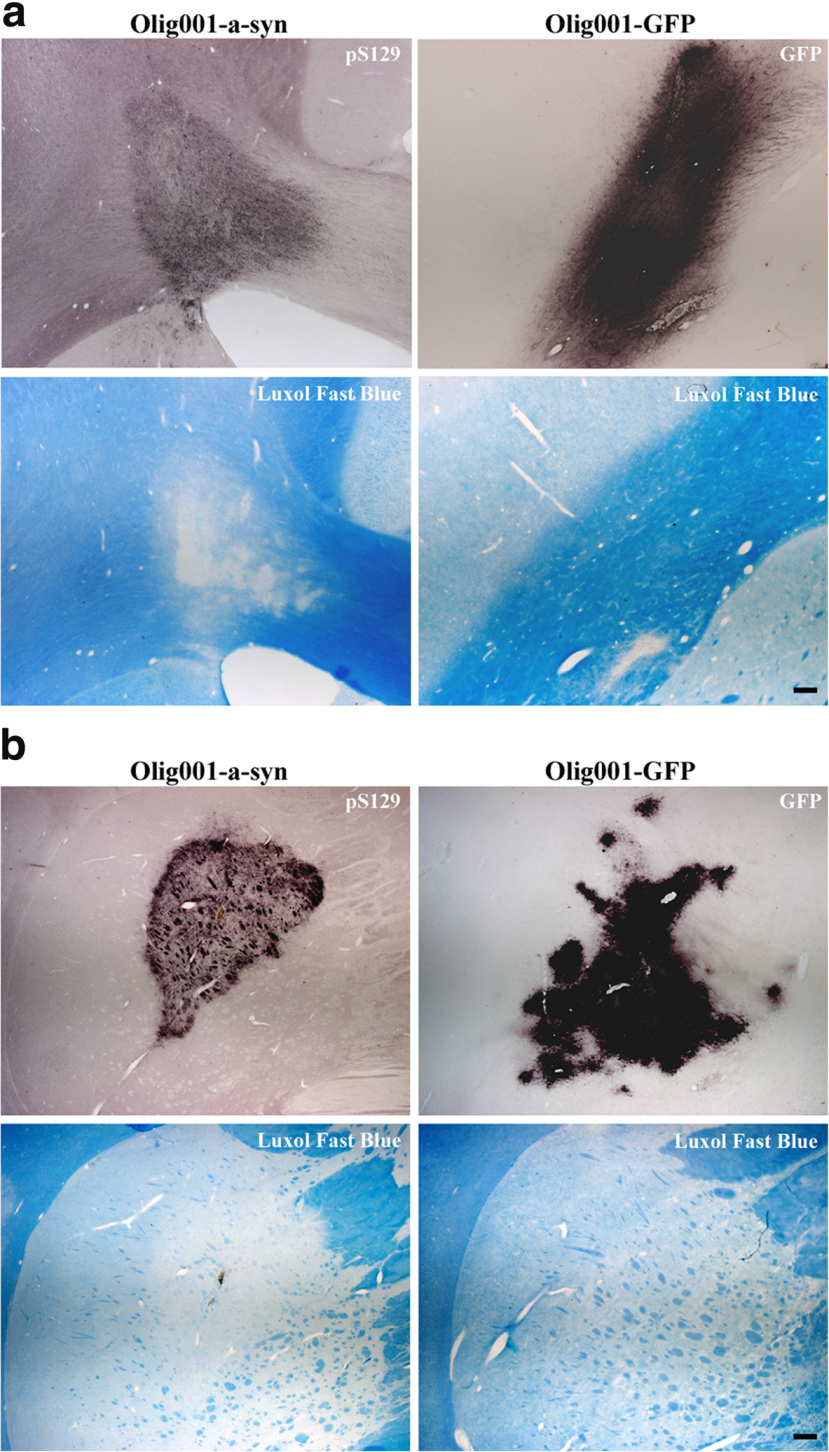
Demyelination in NHPs. Representative images of Olig001-α-syn or Olig001-GFP injected monkeys stained with* Luxol Fast Blue*. **a** Marked demyelination in the corpous callosum of Olig001-α-syn monkeys is shown by decreased* Luxol Fast Blue* staining (*bottom left*). The areas of demyelination correspond to phosphorylated Serine-129 α-syn immunoreactivity in level matched sections (*top left*). No loss of myelin was observed in Olig001-GFP injected monkeys (*bottom right*), indicating that demyelination was a result of α-syn overexpression and not a byproduct of the vector or GFP expression (*top right*). **b** In the striatum, regions of phosphorylated Serine-129 α-syn immunoreactivity (*top left*) associated with robust demyelination of internal capsule fibers and striasomes (*bottom left*), while GFP expression (*top right*) resulted in no changes in myelin (*bottom right*). *Scale bar* 500 μm

Accumulation of GCIs is known to elicit dysfunction of oligodendroglia. Given that oligodendrocytes are responsible for myelination and maintenance of myelin sheaths in the CNS, and loss of myelin is a significant pathological event seen in MSA, we next examined if Olig001-α-syn transduction leads to similar pathology. Luxol Fast Blue was used to visualize myelin in striatal sections of monkeys injected with either Olig001-GFP or Olig001-α-syn. The corpus callosum represents a pure white matter region in which marked demyelination was observed in monkeys injected with Olig001-α-syn (Fig. 4a). Regions of demyelination corresponded to pSer-129 immunoreactivity in level matched sections (Fig. 4a). Conversely, in Olig001-GFP injected monkeys, expression of GFP did not result in demyelination of the corpus callosum (Fig. 4a), indicating that demyelination is specifically related to α-syn, and not a byproduct of the vector or GFP expression. Furthermore, striatal pSer-129 α-syn immunoreactivity in Olig001-α-syn injected NHPs correlated with regions of profound demyelination of striasomes and internal capsule fibers, shown by reduction of Luxol Fast Blue staining in level matched sections (Fig. 4b). Striatal expression of GFP showed no disruption of striatal white matter (Fig. 4b), consistent with the notion that oligodendroglial accumulation of α-syn, but not GFP, causes disruption of oligodendrocyte function.

### Microglial activation in response to Olig001-mediated α-syn overexpression in NHPs

**Fig. 5:**
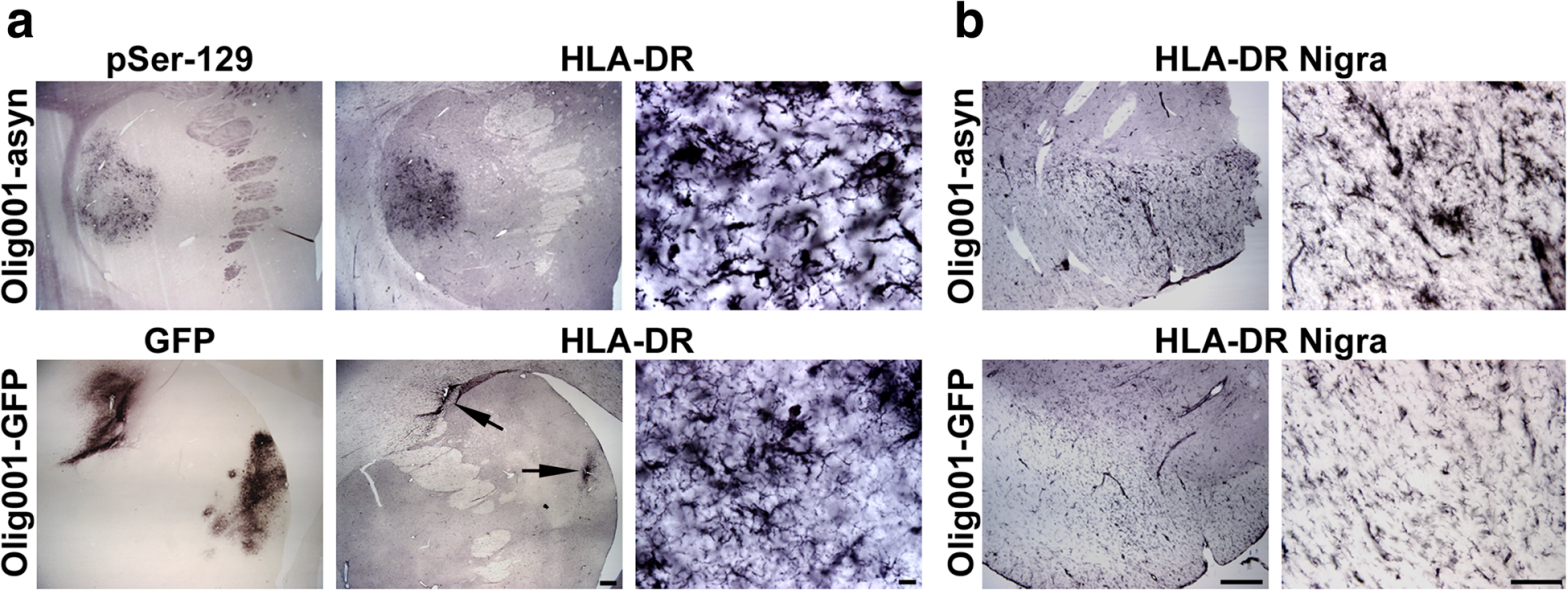
Inflammatory Response **a** Representative striatal images of Olig001-α-syn injected monkeys (*top row*) or Olig001-GFP injected monkeys (*bottom row*). An inflammatory response is seen by HLA-DR+ microglia in white matter tracts of Olig001-α-syn injected monkeys, exclusively covering the entire region corresponding to rAAV- Olig001-mediated α-syn expression. High magnification images show that the morphology of activated microglia. Conversely, in GFP-injected animals, a minimal inflammatory response is seen only in the area of the injection site, not covering the entire region of GFP expression (scale bar 500 μm). High magnification images show that activated microglia are only seen in the immediate vicinity of the injection site (scale bar 10 μm). **b** Activated microglia area also seen in the substantia nigra of Olig001-α-syn injected monkeys, where as the microglia seen in GFP-injected monkeys are in the resting state, shown by a ramified morphology (scale bar low magnification 500 μm, high magnification 100 μm)

Activation of microglia is an early pathogenic process in MSA, often associated with GCI formation. To evaluate the involvement of microglia, monkeys injected with Olig001-GFP and Olig001-α-syn were stained using HLA-DR, which targets MHC class II and serves as a specific marker for activated microglia. In α-syn injected animals, a large inflammatory response was observed throughout the area where the α-syn transgene is expressed, clearly seen in white matter patches of the striatum (Fig. 5a). The morphology of HLA-DR+ microglia clearly indicates an activated state, characterized by a bushy or amoeboid morphology with shrunken processes (Fig. 5a high magnification). In contrast, Olig001-GFP injected animals displayed a minimal inflammatory reaction, limited to the needle tract and specific site of injection (Fig. 5a, injection site marked by arrows). Microglial activation did not cover the entire area of GFP expression, unlike what was observed with α-syn transduction (Fig. 5a). Moreover, activated microglia were seen throughout the substantia nigra of Olig001-α-syn injected monkeys (Fig. 5b). Conversely, in the substantia nigra of Olig001-GFP injected monkeys, microglia were observed to be ramified or “resting” microglia, shown by long-ramified processes with smaller cell bodies (Fig. 5b).

## Discussion

Using silver impregnation (Gallyas method), Papp and Lantos first described the accumulation of insoluble protein aggregates in oligodendrocytes, naming GCIs as the pathological hallmark of MSA [42]. These inclusions were later described to be composed mainly of α-syn [43]. While the precise pathological mechanism(s) remain unknown, the positive correlation between GCI density and neuronal loss highlight the importance of α-syn in the disease process [40]. The drastic demyelination observed in MSA patients is not accompanied by a severe loss of mature oligodendrocytes [49, 14], indicating that accumulation of α-syn leads to dysfunction, rather than overt loss of oligodendroglia. Additionally, alterations in lipid composition are restricted to affected regions in human MSA samples [12]. Moreover, decreased neurotrophic support of oligodendroglia, as shown by reductions of glial derived neurotrophic factor expression levels in MSA tg mice and human MSA samples [56], furthers the idea that oligodendrocyte dysfunction is an early pathological event leading to secondary neurodegeneration related to retrograde axonal disease.

The question of whether α-syn is internalized by oligodendroglia or if it is pathologically overexpressed in oligodendrolia in MSA is still heavily debated, with conflicting reports of elevated α-syn expression [2] and others arguing an absence of elevated α-syn expression in oligodendrocytes in normal individuals [30, 37, 39]. It has been shown that α-syn is transiently expressed in oligodendroglia precursor cells during development, but reduced in mature oligodendrocytes [30, 37, 48]. In contrast, oligodendrocytes differentiated from induced pluripotent stem cells (iPSCs) derived from MSA patients expressed α-syn, whereas iPSCs from PD and healthy controls do not [10] suggesting that the accumulation and aggregation of α-syn in oligodendroglia is specific to the disease process. As such, experimental modeling of MSA critically relies on the overexpression of α-syn in oligodendroglia.

Currently available animal models of MSA are limited to 3 tg mouse lines overexpressing human α-syn under proteolipid protein (PLP) promoter [31], myelin basic protein (MBP) promoter [51], and 2′,3′-cyclic nucleotide 3′-phosphodiesterase (CNP) promoter [67]. Initial studies using PLP-driven expression reported formation of GCIs, however demyelination and neurodegeneration was lacking [31]. Later studies using the same PLP promoter demonstrated subtle motor impairment and a 31.4% loss of nigral neurons [16, 52]. Additional reports of degeneration in non-motor regions of MSA [53], changes in cardiac function [33], and bladder dysfunction [4] have been reported. Mice utilizing CNP-driven overexpression displayed progressive motor impairments and neurodegeneration localized in the spinal cord, with no observed loss in the cerebellum [67]. Overexpression of α-syn using the MBP promoter showed the most classical distribution of pathology, with both the basal ganglia and cerebellum displaying extensive pathology [51]. The degree of GCI accumulation, neurodegeneration, and motor impairments varied significantly with α-syn expression levels, where high expressing lines demonstrated the most significant neuropathological and behavioral deficits [51]. While displaying certain aspects of MSA-like pathology and providing substantial insight of potential disease mechanisms, tg mouse models of MSA harbor inherent limitations. Variability of pathology is seen across the 3 mouse lines, with none of the models being able to model the distinct SND or OPCA observed in MSA patients [3]. Moreover, the constitutive expression of α-syn under oligodendroglia-specific promoters may also incorporate developmentally expressed α-syn in the pathology observed in these models. In support of this, overexpression of α-syn in cell culture models dramatically impaired the maturation of 2 separate oligodendrocyte precursor cell lines, shown by significant reductions of MBP during maturation [13].

The variable pathology and potential problem of constitutively expressing α-syn in tg mouse models, along with the lack of rodent and primate models of MSA, lead us to utilized a novel oligodendrocyte-directed AAV capsid, Olig001 [44], in order to develop a viral vector based model of MSA. Olig001 was developed using capsid shuffling and directed evolution, resulting in a chimeric capsid composed of AAV1, 2, 6, 8, and 9, which exhibits oligo-specific tropism 9 fold higher than wild-type AAVs [44]. The high level of oligodendroglia tropism allowed transgene expression to be driven by a constitutive chicken beta-actin hybrid (CBh) promoter. Previous studies by other groups target oligodendroglia using wild-type rAAVs with expression driven by MBP promoters, however the overall transduction of oligodendrocytes was still low [8, 34]. In the present study, intrastriatal injection of Olig001-GFP in rats successfully transduced oligodendroglia 94–97% of the time, with less than 5% off-target transduction of either neurons or astrocytes, demonstrating a 19:1specificity for oligodendrocytes.

With translational studies in mind, it is important to demonstrate consistent tropism of a particular rAAV, as examples where the tropism of a given rAAV capsid does not remain consistent in rodents and higher animals have been described [7]. In the current study, we observed that the affinity of Olig001 to transduce oligodendroglia was conserved between species. Intrastriatal injection of Olig001-GFP in rhesus macaques demonstrates the same preference for oligodendrocytes as in rodents [F(1,5) = 3.4, *p* > 0.12]. Furthermore, there was slightly less co-localization of GFP and NeuN in primates than in rodents [3.7% rodents vs. 0.7% primates (F1,5) = 31.7, *p* = 0.002], indicating that Olig001 produced less off target transduction in nonhuman primates.

Given the high level of specificity of Olig001 for oligodendroglia in both rats and primates, we transduced rhesus macaques with Olig001 containing the α-syn transgene in an attempt to develop the first nonhuman primate model of MSA. Intrastriatal injection of Olig001-α-syn produced widespread expression of α-syn throughout white matter regions of the striatum. Inclusions of α-syn were found to localize inside oligodendroglia, shown by co-localization of LB509 and oligodendroglia marker Olig2. Furthermore, the inclusions were phosphorylated at Serine-129 and resistant to proteinase K digestion, recapitulating many characteristics of GCIs found in human MSA samples.

Early pathological changes seen in MSA were demonstrated in the monkeys 3-months following transduction. Most notably, marked demyelination was observed throughout striatal white matter, demonstrated by reduced Luxol Fast Blue staining in the corpous callosum and striatal white matter bundles. Importantly, the observed demyelination was due to the accumulation of α-syn in oligodendroglia, as areas of pSer-129 immunoreactivity highly correlated with regions of myelin loss. Moreover, this was not a result of the AAV, as no reductions in myelin were observed in the Olig001-GFP transduced animals. While the differing post-injection time course of the Olig001-GFP and Olig001-α-syn injected monkeys does not allow for a perfect comparison, there is an enormous amount of data from numerous NHP studies following striatal injection of different transgenes that never reported demyelination or α-syn aggregation. Luxol Fast Blue and pSer-129 staining of rhesus macaques 3-months after receiving intrastriatal injections of AAV2-neurturin [25] or stem cells, as well as untreated animals, displayed no demyelination or aggregated α-syn which supports the specificity of effects observed with Olig001-GFP and Olig001-α-syn. In line with post-mortem human MSA cases, the demyelination observed in our model is not associated with any obvious reductions in oligodendroglia, further indicating that the loss of myelin is a result of oligodendrocyte dysfunction and not an overt loss of oligodendroglia.

Our model recapitulated other pathological events observed in MSA, such as immune and inflammatory activation. It has been reported that activated microglia are found within white matter tracts and areas of neurodegeneration in patients with MSA, and are believed to release proinflammatory cytokines and possibly play a role in neurodegeneration [26, 53]. Widespread HLA-DR+ activated microglia are seen covering α-syn transduced areas of striatal white matter, whereas minimal activation was seen specifically at the injection site in GFP transduced monkeys. Activated microglia were also observed in the substantia nigra of α-syn transduced monkeys, whereas a ‘non-activated phenotype was observed following GFP transduction. In our previous work, we have seen that a capsid and/or transgene related inflammatory response peaks at 2-weeks post-injection and then retracts to the needle track [46], similarly to what is seen with the Olig001-GFP injected monkeys. Importantly, this suggests that the inflammatory response seen in monkeys 3-months after injection of Olig001-α-syn is directly related to virally delivered α-syn expression, and not an ongoing inflammatory response due to the rAAV injection.

This model is presently ripe for replication and extension. Due to the exploratory nature of our initial experiment, we only examined the effects of Olig001 gene delivery over a short post-surgical time course. We are currently studying longer time courses and hypothesize that neurodegeneration would occur later than 3-months after administration of our vector. The accumulation of activated microglia in demyelinated areas of the striatum and in the substantia nigra could be the pathological event immediately preceding neuronal loss. Many groups have proposed the hypothesis that accumulation of α-syn in oligodendroglia leads to loss of myelin, and that the now unmyelinated axons are exposed and vulnerable to proinflammatory cytokines released by activated microglia seen in the vicinity of GCIs. These early pathologic events, with the addition of reduction of neurotrophic support, lead to axonal damage and subsequent cell death, implicating MSA as a retrograde axonal disorder. Our model recapitulates early pathogenesis seen in MSA, as many of the pathological features induce dysfunction of the oligodendroglia-myelin-axon-neuron complex [28].

## Conclusions

In conclusion, our findings provide evidence that viral vector-mediated overexpression of α-syn can transduce oligodendroglia specificially as seen in MSA and reproduces many of the early pathologic features of this disease, indicating the development of the first ever nonhuman primate model of Multiple System Atrophy. Further studies are needed to assess the potential of this model to develop behavioral impairments and neurodegeneration, however, this model could prove useful for elucidating the precise pathological mechanism of MSA. Furthermore, future studies using this model may provide evidence that MSA is a primary oligodendrogliopathy, in that accumulation of α-syn in oligodendroglia results in demyelination prior to neuronal loss. Additionally, NHP disease models are extremely important for pre-clinical testing of experimental therapeutics, as success in mouse models of MSA have not translated well for the treatment of the human disorder.

## Additional files

Additional file 1: Figure S1. Z-stack confocal images of Olig001-GFP injected rats verifies the colocalization of GFP (green) with oligodendroglia marker Olig2 (red), indicating that the vector is in fact transducing oligodendrocytes. (*Scale bar* 20 μm) (TIFF 3123 kb)

Additional file 2: Figure S2. Z-stack confocal images of Olig001-GFP injected nonhuman primates verifies the colocalization of GFP (green) with oligodendroglia marker Olig2 (red), indicating that the vector is in fact transducing oligodendrocytes in NHPs. (*Scale bar* 20 μm) (TIFF 3145 kb)

Additional file 3: Figure S3. Phenotypic comparison of transduced cells across species shows no differences in oligodendrocyte-specific tropism of Olig001-GFP 4-weeks following injection. (TIFF 40 kb)
