# Supplementary figures and images for: Novel oligodendroglial alpha synuclein viral vector models of multiple system atrophy: studies in rodents and nonhuman primates
Source: Acta Neuropathol Commun. 2017 Jun 16;5:47. doi: 10.1186/s40478-017-0451-7 (PMC5473003; doi:10.1186/s40478-017-0451-7)

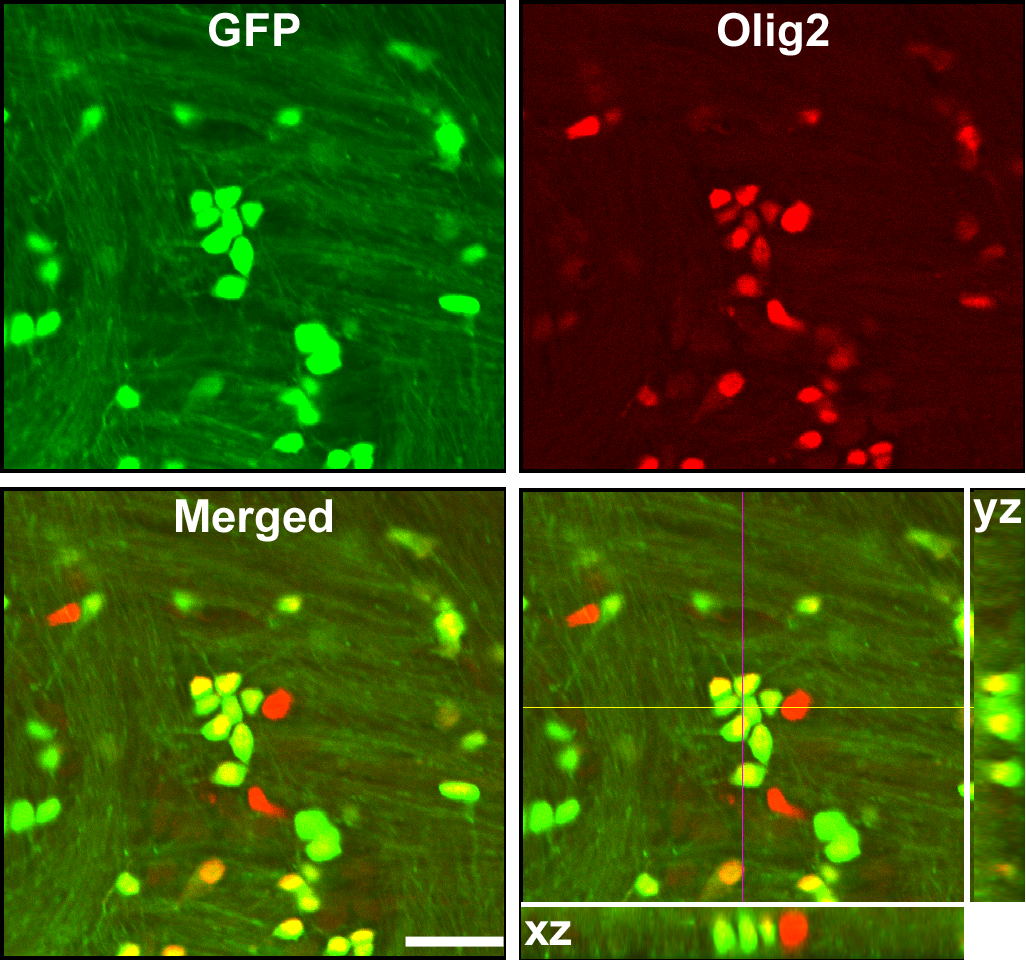

Supplement: Supplementary file 1 — Z-stack confocal images of Olig001-GFP injected rats verifies the colocalization of GFP (green) with oligodendroglia marker Olig2 (red), indicating that the vector is in fact transducing oligodendrocytes. (Scale bar 20 μm) (TIFF 3123 kb) [file 40478_2017_451_MOESM1_ESM.tif]

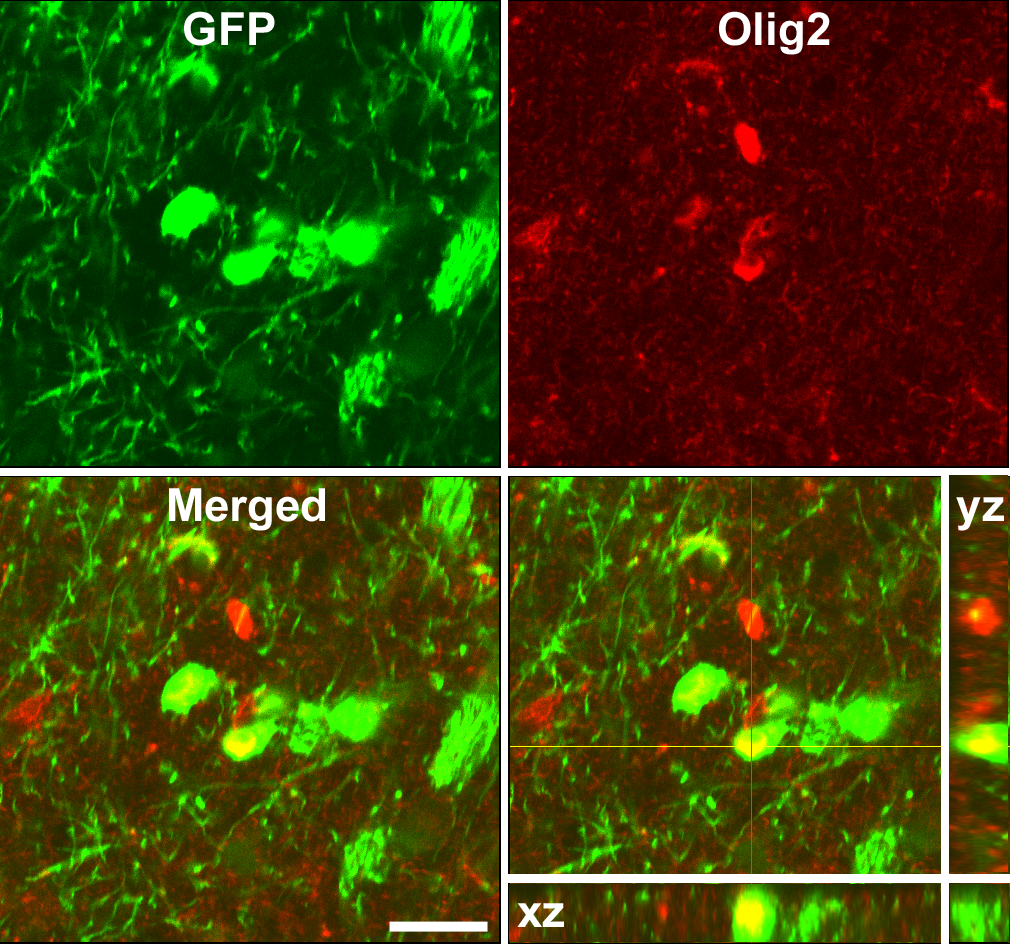

Supplement: Supplementary file 2 — Z-stack confocal images of Olig001-GFP injected nonhuman primates verifies the colocalization of GFP (green) with oligodendroglia marker Olig2 (red), indicating that the vector is in fact transducing oligodendrocytes in NHPs. (Scale bar 20 μm) (TIFF 3145 kb) [file 40478_2017_451_MOESM2_ESM.tif]

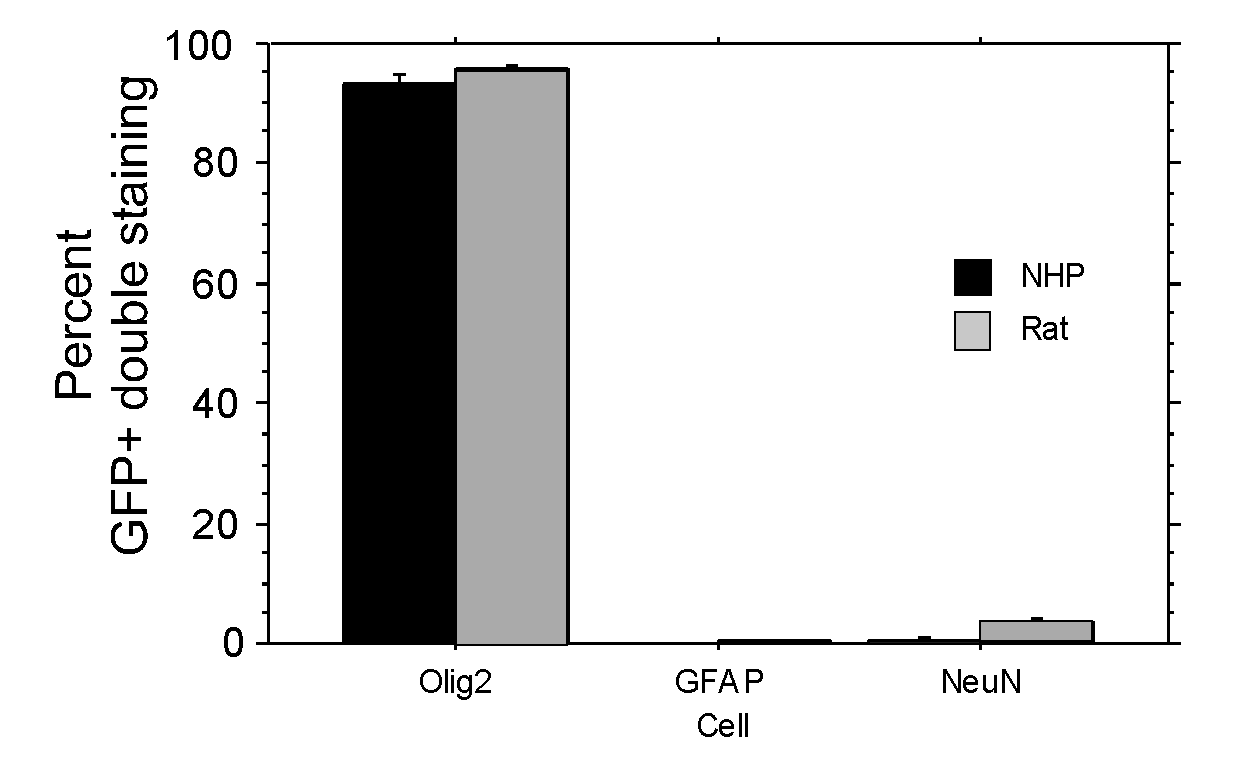

Supplement: Supplementary file 3 — Phenotypic comparison of transduced cells across species shows no differences in oligodendrocyte-specific tropism of Olig001-GFP 4-weeks following injection. (TIFF 40 kb) [file 40478_2017_451_MOESM3_ESM.tif]
